# Editorial: Real-World Evidence of Pediatric Exposure to Psychopharmacologic Medications

**DOI:** 10.3389/fpsyt.2022.930276

**Published:** 2022-06-10

**Authors:** Julie M. Zito, Susan DosReis, Bruce Carleton

**Affiliations:** ^1^Department of Pharmaceutical Health Services, University of Maryland, Baltimore, MD, United States; ^2^Department of Psychiatry, School of Medicine, University of Maryland, Baltimore, MD, United States; ^3^Department of Pediatrics, Division of Translational Therapeutics, Faculty of Medicine, The University of British Columbia, Vancouver, BC, Canada

**Keywords:** real-world evidence (RWE), pharmacoepidemiology, psychopharmacologic medications, children, youth, psychopharmacologic drugs, adolescents

This Research Topic on “Real-world evidence of pediatric exposure to psychopharmacologic medications” comprises nine invited studies reflecting the state of the art in pharmacoepidemiologic research circa 2022. From Europe and North America, these studies offer real-world data (RWD) on pediatric medication practices. In many cases, concerns are raised around how young people are treated (population-based prevalence) and user characteristics. These concerns relate to safety surrounding off-label use due to age or indications, inter-class polypharmacy, risk of long-term pediatric use, and fetal exposure. Two examples, based on follow-up studies, illustrate poor patient monitoring for treatment-emergent events. Five international studies are briefly described below, followed by four US studies.

A Norwegian study conducted by Kiselev et al. on the prevalence of autism spectrum disorders among 2–17-year-olds reaffirms the value of a national registry in assessing the prevalence of the disorder and its psychotropic treatment. The findings indicate a 2014 prevalence of 0.76% of ASD in Norway and the co-occurrence of comorbid diagnoses account for the use of psychotropic drug class use. Comparing their findings to a 2.24% prevalence in young populations from the US, they indicate a far greater ASD prevalence and drug treatment in the U.S. than in Norway. Researchers cooperating across the US—Europe divide have been showing us similar disparities for more than a decade (1).

Following our metaphorical journey through the five international studies, we journey south from Norway to the Netherlands where Minjon et al. examine electronic health records (EHR). The authors aim to bring enhanced information from *new* atypical antipsychotic (AAP) users, i.e., with no prior AAP use for 6 months. A 3-year follow-up was conducted in community-based psychiatric clinics. The outcomes consist of a frequency of reports of physical measures (e.g., weight, pulse, blood pressure) and laboratory parameters (e.g., glucose and triglycerides) at baseline and in subsequent 6-month intervals. The results reveal low frequencies in both physical and laboratory monitoring. While it is exciting to see EHRs are now available for closer, more accessible monitoring of health care, the results suggest the benefit does not necessarily extend to improved prescriber compliance, with recommendations for safer use of second-generation antipsychotics.

In nearby Germany, Scholle et al. were able to capitalize on the continuity of care from universal healthcare coverage. In this context, the study was able to provide a 4-year baseline from which to identify new users of ADHD medication. The rich dataset enabled a robust analysis of conformance to clinical prescribing standards.

Across the pond in Canada, Gober et al. extracted British Columbia data across 20 years to assess preschoolers (0–5 years) on the relationship of hydroxyzine exposures to subsequent mental health diagnoses in a follow-up to age 10. Young patients with 5 or more hydroxyzine dispensings were significantly more likely to have a diagnosis of tic disorder compared with those receiving one dispensing [odds ratio = 1.40 (1.08–1.81)]. Trends for anxiety and emotional disturbance were also significant. The context of this study suggests it is a “hot topic,” and that confirming clinical trial evidence could bring substantial change to current pediatric practice.

Campbell et al. offer a unique example of a Canadian clinical study to assess fetal factors associated with SSRI antidepressant use in 148 pregnant women. Fetal heart rate and heart rate variability (HRV) were assessed at 36-weeks' gestation among 4 groups of pregnant women. While heart rate was not significantly different, HRV was significantly reduced in SSRI-non-depressed exposed male but not female fetuses. The effect increased with higher SSRI dosage. HRV changes were within the normal range of developing fetuses at 36-weeks' gestation, suggesting the effects are not likely of clinical significance. While the authors urge replication, readers of this series on real-world evidence may be reminded of the widespread use of SSRIs among young people as well as pregnant women and the growing concerns about the questionable effectiveness (2) and difficulty withdrawing from SSRIs (3).

Among US research studies, Zhang et al. assessed U.S. data on young people who were commercially insured to extend the stimulant cardiovascular safety question to the increasingly common use of concomitant stimulants and atypical antipsychotic (S+AAP). A time-dependent logistic model calculated the less severe event risk (LSE) for current concomitant S+AAP users compared with past users and non-users. LSE risks appear modest but consistent: 14 LSE per 10,000 person months (p-m) for current users and 8.2 per 10,000 p-m for past users, compared with non-users, respectively. The risk for combination S+AAP was not statistically significant. Nevertheless, the search for safety data on frequently occurring off-label combinations, such as stimulants and atypical antipsychotics, will no doubt continue, especially since commercial datasets reveal relatively short exposures and modest AAP dosage.

Prescribers in recent years have learned of “deprescribing,” a term created to address multidrug regimens that may exceed the benefit of combinations, which are mostly off-label and suggest discontinuation. To fill the gap in clinical practice protocols for safe pediatric withdrawal of stimulants, Lohr et al. conducted a systematic review on clinical practices to safely discontinue stimulants for young people treated for ADHD. After a close review of 35 studies, with several clinical trials among them, a subgroup was identified for whom relapse or deterioration did not occur following discontinuation of stimulants. Approximately 30% of stimulant trial participants were found to support community treatment efforts to discontinue stimulants in young people who did not benefit from them.

Deprescribing is again called for by Edelsohn et al. in their U.S. analysis of the concomitant psychotropic prescribing patterns from admission to discharge in residential care. Logistic regression showed that among patient and treatment characteristics only the number of medications prescribed at admission was significant (*p* < 0.001), with more medications at admission contributing to the probability of discharge on four or more concomitant psychotropics.

Further support for safe prescribing protocols can be surmised from the systematic review of U.S. studies of inter-class polypharmacy by Zito et al. (Full disclosure: Zito is the first author of this review). The growth of 3 or more psychotropic class polypharmacy is confirmed and currently, more than 300,000 U.S. polypharmacy medicated young people (4) could benefit from reduced or discontinued medication. Here, as in Lohr et al. and Edelsohn et al., the evidence calls for deprescribing research as well as post-marketing research to establish the effectiveness, safety, and tolerability of complex concomitant regimens in community populations, e.g., in large simple trials.

Collectively, these pharmacoepidemiologic real-world studies reiterate the need for future post-marketing drug studies to assure us that widely used off-label psychopharmacologic agents are beneficial and safe. Perhaps it is time to seek research funds to measure population-based outcomes in sufficient detail (functioning, social development) to assure that the benefits of pediatric psychotropics outweigh the risks.

## Author Contributions

JZ drafted the manuscript. SD and BC reviewed and edited the manuscript. All authors contributed to the article and approved the submitted version.

## Conflict of Interest

The authors declare that the research was conducted in the absence of any commercial or financial relationships that could be construed as a potential conflict of interest.

## Publisher's Note

All claims expressed in this article are solely those of the authors and do not necessarily represent those of their affiliated organizations, or those of the publisher, the editors and the reviewers. Any product that may be evaluated in this article, or claim that may be made by its manufacturer, is not guaranteed or endorsed by the publisher.
